# Standardized Full-Field Electroretinography in the Green Monkey (*Chlorocebus sabaeus*)

**DOI:** 10.1371/journal.pone.0111569

**Published:** 2014-10-31

**Authors:** Joseph Bouskila, Pasha Javadi, Roberta M. Palmour, Jean-François Bouchard, Maurice Ptito

**Affiliations:** 1 School of Optometry, University of Montreal, Montreal, Quebec, Canada; 2 Biomedical Sciences, Faculty of Medicine, University of Montreal, Montreal, Quebec, Canada; 3 Behavioral Science Foundation, Basseterre, St. Kitts, West Indies; 4 Departments of Psychiatry and Human Genetics, McGill University, Montreal, Quebec, Canada; 5 BRAINlab and Neuropsychiatry Laboratory, Department of Neuroscience and Pharmacology, University of Copenhagen, Copenhagen, Denmark; University of Houston, United States of America

## Abstract

Full-field electroretinography is an objective measure of retinal function, serving as an important diagnostic clinical tool in ophthalmology for evaluating the integrity of the retina. Given the similarity between the anatomy and physiology of the human and Green Monkey eyes, this species has increasingly become a favorable non-human primate model for assessing ocular defects in humans. To test this model, we obtained full-field electroretinographic recordings (ERG) and normal values for standard responses required by the International Society for Clinical Electrophysiology of Vision (ISCEV). Photopic and scotopic ERG recordings were obtained by full-field stimulation over a range of 6 log units of intensity in dark-adapted or light-adapted eyes of adult Green Monkeys (*Chlorocebus sabaeus*). Intensity, duration, and interval of light stimuli were varied separately. Reproducible values of amplitude and latency were obtained for the a- and b-waves, under well-controlled adaptation and stimulus conditions; the i-wave was also easily identifiable and separated from the a-b-wave complex in the photopic ERG. The recordings obtained in the healthy Green Monkey matched very well with those in humans and other non-human primate species (*Macaca mulatta* and *Macaca fascicularis*). These results validate the Green Monkey as an excellent non-human primate model, with potential to serve for testing retinal function following various manipulations such as visual deprivation or drug evaluation.

## Introduction

The retina is a complex and well-organized neuronal structure that is vulnerable to internal influences such as retinopathies and ocular pathologies, and is furthermore sensitive to external factors such as drugs and alcohol toxicity. Full-field electroretinography represents a useful diagnostic clinical tool in ophthalmology and is widely used as a measure of retinal function. Electroretinogram (ERG) recordings are generated through different summation of currents evoked in distinct populations of retinal cells, including photoreceptors (cones and rods), neurons (horizontal cells, bipolar cells, amacrine cells, and ganglion cells), glial cells (Müller cells), and epithelial cells [1]. Accordingly, the influence of environmental manipulations on the function of retinal cells can be assessed objectively in the ERG. Whereas the full-field ERG reflects the response of the entire retina to stimulus, it is possible to differentiate between responses of various retinal structures to light [2]; in fact, the positive and negative waves of the ERG emerge from different levels of retinal processing, and the response of particular retinal cell populations and circuits is target by the choice of stimulus and recording environment. Research on the origins of pathophysiological conditions displayed in human electroretinography is mostly carried out in animal models, with non-human primates remaining particularly important in visual neuroscience research, due to their superior emulation of human retinal function [3]. Indeed, the non-human primate ERG plays an important role in studies of visual abnormalities and potentially therapeutic pharmacological effects in the retina. The International Society for Clinical Electrophysiology of Vision (ISCEV) proposes a minimum of five types of measurements in order to obtain standardization for investigations in humans [4], all of which can be obtained in non-human primates.

Green Monkeys have become important non-human primate species for visual neuroscience research. The genome of Green Monkeys has 90% parity with the human genome, which lends support to its use to model a range of behavioral and non-behavioral pathologic disorders in human [5], [6]. In fact, Green Monkeys are used as a model organism for the study of diabetes, cardiovascular disease, HIV/AIDS, Parkinson's disease, substance abuse, attention deficit disorder, alcoholism, reproduction, tissue regeneration and other conditions [5], [7], [8], [9]. The Green Monkey has been utilized in visual neuroscience for many years [7], [10], [11], [12], [13], [14], [15], [16], [17], [18], [19], leading to a thorough anatomical description of the visual pathways and the publication of anatomical brain atlases [20], [21], [22]. Their large brain and ocular size relative to the 3.5 kg bodyweight of adult Green Monkeys is particularly advantageous in the electrophysiological study of visual abnormalities arising in the retina and optic nerve. The organization of the retina of the green monkey is similar to that of other Old World species such as Macaques, for example. The retina contains several layers and different cell populations: photoreceptors, bipolar cells, ganglion cells, amacrines and horizontal cells. There is a monotonic decrease in the number of cones from the fovea centralis (containing mainly cones) to the periphery made out of rods [17], [23]. This developed fovea is well suited for high visual acuity, color vision and photopic sensitivity whereas the peripheral retina is responsible for scotopic vision (nocturnal) [24]. From the study of Herbin et al. (1997), the only one available on the green monkey retina, the retinal ganglion cells (RGCs) number derived from retinal wholemounts was estimated at 1 228 646. The topographical distribution of RGCs shows a strong centro-peripheral gradient, with the majority of small cells (P cells) in the fovea, the larger ones being encountered in the periphery (M cells). The axons of the ganglion cells form the optic nerve and their counts derived from semi-thin sections (1 220 000) are close to the estimated number of RGCs for the vervet monkey and are in the range with those reported for *Macaca Mulatta* (1 468 000 RGCs) [25].

However, little is known about the electrophysiology of the Green Monkey retina, since most of such studies have been conducted in the rhesus monkey, for which a standardized procedure for electroretinographic examination has been published [26]. Due to the lack of corresponding data in Green Monkeys despite their growing importance in visual neuroscience, a standardized electroretinography protocol is needed. We therefore present here full-field ERG data for Green Monkeys, including the five standard responses recommended by the ISCEV.

## Materials and Methods

### Animals

A total of 15 adult male and female Green Monkeys (*Chlorocebus sabaeus*), aged 3 to 4 years and weighing 3.01±0.35 Kg, were used for this study (Table 1). The animals were born and raised in enriched environments in the laboratories of the Behavioral Science Foundation (St-Kitts, West Indies). As adults, the animals were fed with primate chow (Harlan Teklad High Protein Monkey Diet; Harlan Teklad, Madison, WI) and fresh local fruits, with water available *ad libitum*. Infant Green Monkeys are born into an outdoor social group comprising several females, one male and other offspring of the same general age. Infants live with their parents until about 8 months of age, at which time they move to a playpen with 5 other age-mates. The natal cage is equipped with swings, perches, hiding places and jungle gyms. We do put in toys, but the animals are so busy playing with one another that they ignore the toys. In the smaller playpens, there are also swings, perches and climbing spots, as well as puzzle feeders and foraging boards. At about 18 months of age, youngsters graduate to a large, outdoor peer group of about 16 animals (like-ages, both sexes) where there are tunnels, swings, ladders, jungle-gyms and a variety of manipulanda (more complex puzzle feeders; natural forage opportunities, such as brush and vines; foraging boards). Plastic chain and baited balls are popular toys, but vervets of this age are uninterested in most other commercially available toys. All experiments were performed according to the guidelines of the Canadian Council on Animal Care (CCAC) and the Association for Research in Vision and Ophthalmology (ARVO) Statement for the Use of Animals in Ophthalmic and Vision Research. The experimental protocol was also reviewed and approved by the local Animal Care and Use Committee (University of Montreal, protocol # 14-007) and the Institutional Review Board of the Behavioral Science Foundation that is recognized by the CCAC. None of the animals were sacrificed for this study.

**Table 1:** Subject profile of animals used in this study.

	Animal ID	Sex	Weight (Kg)	IOP (mm Hg)	Pupil dilatation (mm)
**1**	05011-5	Male	3.950	OD 10/OS 9	OD 9/OS 9
**2**	05010-6	Male	3.725	OD 7/OS 7	OD 8/OS 8
**3**	09093-1-3-1	Female	3.050	OD 9/OS 9	OD 9/OS 9
**4**	08274	Female	2.800	OD 10/OS 11	OD 9/OS 9
**5**	08275	Female	2.925	OD 8/OS 6	OD 8.5/OS 8.5
**6**	07862	Female	2.875	OD 15/OS15	OD 8.5/OS 8.5
**7**	08297	Female	2.750	OD 6/OS 6	OD 9/OS 9
**8**	07866	Female	2.950	OD 12/OS 12	OD 9/OS 9
**9**	01336-7-1-3	Female	2.950	OD 12/OS 13	OD 9/OS 9
**10**	08315	Female	2.775	OD 10/OS 11	OD 9/OS 9
**11**	07868	Female	2.825	OD 8/OS8	OD 8.5/OS 8.5
**12**	08375	Female	2.900	OD 11/OS 12	OD 9/OS 9
**13**	08376	Female	2.850	OD 6/OS 6	OD 9/OS 9
**14**	08336	Female	2.925	OD 14/OS 15	OD 9/OS 9
**15**	08377	Female	2.852	OD 9/OS 10	OD 9/OS 9

### Animal preparation for ERG recording

**Figure 1:**
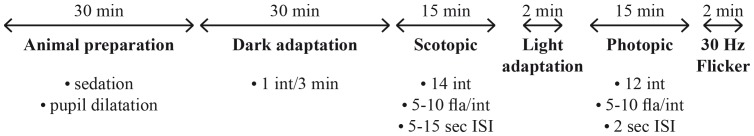
Summarized schematic procedure describing a typical electroretinography recording session in a Green Monkey (*Chlorocebus sabaeus*). Int, intensity; Fla, flashes; ISI, inter stimulus interval.

The following procedure describes a typical recording session in Green Monkeys, including successively a 30 minutes of animal preparation, 30 minutes of dark adaptation, 15 minutes of scotopic recordings, 2 minutes of light adaptation, 15 minutes of photopic recordings, and 2 minutes of flicker recordings (Figure 1). The values of dark and light adaptation were chosen based on data obtained in cynomologus monkeys [27]. The animals were sedated with an intramuscular injection of a mixture of ketamine (10 mg/kg; Troy Laboratories, Glendenning, New South Wales, Australia) and xylazine (1 mg/kg; Lloyd Laboratories, Shenandoah, IA). In this condition, the pupils were fully dilated to approximately 9 mm in diameter and the accommodation reflex was paralyzed with topical application of 1% tropicamide (Mydriacyl) and 2.5% phenylephrine hydrochloride (Mydfrin) (Alcon Laboratories, Fort Worth, TX). Intraocular pressures (IOP) were also monitored before and after the recording session by applanation tonometry (TonoPen XL; Mentor, Norwell, MA, USA). There were no significant IOP and pupil size differences noted between the beginning and the end of the ERG procedure. The eyes were treated with 0.5% proparacaine hydrochloride (Alcaine; Alcon Laboratories, Fort Worth, TX, USA) to anesthetize the cornea and then protected by application of 2.5% methylcellulose (Gonak; Akorn, Inc., Buffalo Grove, IL, USA) to prevent corneal drying. Body temperature was maintained between 36.5°C and 38°C with a heating pad. Recording sessions lasted approximately two hours for each animal, after which they were allowed to recover and returned to their prior naturalistic setting.

### Visual Stimulation

Full-field stimulation was produced with an UTAS BigShot Ganzfeld light source (UTAS E-3000 electrophysiology equipment; LKC Technologies, Inc., Gaithersburg, MD, USA) that was placed in front of the animal's face. Both eyes were simultaneously recorded and averaged as detailed below. The ERGs were evoked by white flashes of light of intensities ranging from 0.00025 cd.sec.m^−2^ to 1000 cd.sec.m^−2^ delivered in full-field conditions. During the course of dark adaptation, ERGs were recorded at 3 minutes intervals over 30 minutes of dark adaptation with a constant stimulus of approximately 0.025 cd.s.m^−2^. LED flash luminance of 0.00025 to 6 cd.sec.m^−2^ (−50 dB to 4 dB in LKC units) was used for scotopic stimulation. Responses were averaged for each of the 14 time-integrated flash luminance levels presented (ranging from −3.6 to 2.9 log cd.s.m^−2^ in approximately 0.3 log-unit steps; flash duration, 20 µs; inter-stimulus interval, 5 sec for −3.6 to 0.4 log cd.s.m^−2^ and 15 sec for 0.6 to 2.9 log cd.s.m^−2^) and xenon flash luminance of 2.5 to 800 cd.sec.m^−2^ (0 dB to 25 dB in LKC units) for photopic stimulation (ranging from −2.2 to 2.9 log cd.s.m^−2^ in approximately 0.3 log-unit steps; flash duration, 20 µs; inter-stimulus interval, 2 sec for all intensities). For light-adapted ERGs a steady white background-adapting field (30 cd/m^2^) was presented inside the Ganzfeld to saturate the rod system. Flash intensities and background luminance were calibrated using a research radiometer (IL1700 Photometer; International Light Inc., Newburyport, MA, USA) with a SED033 detector placed at 36 cm from the source.

### ERG recording and analysis

All experimental protocols followed the guidelines of the ISCEV [4], specifying the 5 standard responses: (1) a dark-adapted response (rod response), (2) a dark-adapted maximal response (combined rod–cone response), (3) a dark-adapted oscillatory potentials response, (4) a light-adapted response (cone response), and (5) a light-adapted response to a rapidly repeated stimulus (30 Hz flicker). ERG recordings and signal processing were recorded with contact lens electrodes lying across the center of the cornea of each eye moistened with 1% carboxymethylcellulose sodium (Refresh Celluvisc, Allergan Inc., Markham, ON, Canada). The corneal contact lens electrode (Jet electrodes; Diagnosys LLC, Lowell, MA, USA) was equipped with four small posts on the convex surface in order to keep the eyelids open. Reference and ground gold disc electrodes (model F-E5GH; Grass Technologies, Astro-Med, Inc., West Warwick, RI, USA) were kept in place with adhesive paste (Ten20 conductive EEG paste; Kappa Medical, Prescott, AZ, USA) at the external canthi and forehead, respectively. Responses were amplified 10,000 times and filtered with a band pass from 1 to 500 Hz except for the oscillatory potentials, which were extracted with the LKC software with a band pass from 75 to 500 Hz. Each tracing included a 20 ms pre-stimulus baseline. Depending on the measured stimulus, up to 10 waveforms were averaged to reduce variability and background noise. Based on literature focusing on the origins of ERG waves in a primate model (macaque monkey) whose retina is very similar to that of humans [2], the origins of the waveforms are described. For the waveform analysis, the amplitude of the a-wave, which mainly reflects the function of photoreceptors, was measured from the baseline to the peak of the a-wave for the combined rod-cone response and the single-flash cone response. The amplitude of the b-wave, which reflects the activity of the inner nuclear layer, was measured from the peak of the a-wave to the peak of the b-wave for all responses. The peak latency was defined from the onset of the flash to the peak. In the case of the oscillatory potentials, the latency to the second peak was usually determined, where the amplitude was defined as peak to trough amplitude from the peak of the second wave to the following trough. The amplitude of the i-wave was measured from the trough of the b-wave to the peak of the i-wave and its respective peak time was also measured from flash onset. The exact origin of the i-wave is still controversial. Some have suggested that this component is generated at the inner retinal level [28], and others that it origins at a more distal location [29]. The latter point is highlighted in ocular pathology studies. For example, the i-wave in glaucoma patients [30] and in glaucoma animal models [29] is increased, suggesting that it does indeed originate in the distal retina. In the 30 Hz flicker ERG, the second peak was evaluated in relation to the preceding trough. Retinal response diagrams were drawn using Adobe Illustrator and processed in Adobe InDesign (Adobe Systems, software version CS5; San Jose, CA, USA). The ERG procedure is summarized schematically in Figure 1.

## Results

### The five standard responses

**Figure 2:**
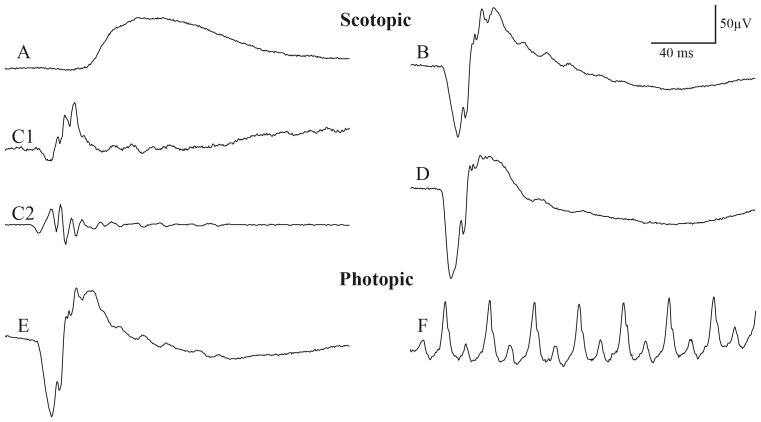
Standard responses for full-field electroretinography in a representative Green Monkey (*Chlorocebus sabaeus*), including the 5 standard responses: a rod response, a combined rod–cone response, oscillatory potentials, a cone response, and a flicker response. (**A**) Rod response elicited at −2.2 log cd.s.m^−2^ (0.0064 cd.s.m^−2^) after 30 minutes of dark adaptation. (**B**) Maximal response elicited at 0.4 log cd.s.m^−2^ (2.57 cd.s.m^−2^, standard flash) in the dark-adapted eye. (**C1**) Broadband scotopic ERG waveform and (**C2**) the corresponding software-filtered oscillatory potentials elicited at 0.6 log cd.s.m^−2^ (4.4 cd.s.m^−2^) in the dark-adapted eye. (**D**) The recommended additional stronger flash ERG elicited at 10.0 cd.s.m^−2^ in the dark-adapted eye. (**E**) White flash cone response elicited at 0.4 log cd.s.m^−2^ in the light adapted eye with a background illumination of 30 cd.m^−2^. (**F**) Flicker response (30 Hz) elicited at 0.4 log cd.s.m^−2^ after five minutes of light adaptation with a background illumination of 30 cd.m^−2^. Tracings (**A**, **B**, **C1**, **D**, **E**) included a 20 ms pre-stimulus baseline. Horizontal calibration, 40 ms; vertical calibration, 50 µV.

All 15 Green Monkeys displayed very well detectable and easily reproducible ERG recordings, using the protocol described in the Experimental Procedure section (Figure 1). As indicated by the ISCEV, the five standard responses and the recommended additional stronger scotopic flash ERG are illustrated in Figure 2 for a representative Green Monkey. The typical scotopic ERG signal is formed, as expected, by an initial negative wave (the a-wave) and followed by a larger positive wave (the b-wave). Faster components of lower amplitude, known as the oscillatory potentials (OPs), are seen in the ascending limb of the scotopic b-wave. These OPs were as prominent as those obtained in humans. Given uncertainty of how best to quantify OPs, we chose to measure the amplitude from the peak of the second wave to the following trough, as described in the Experimental Procedure section. Thus, the chronological sequence of electrical events in a typical photopic ERG response observed in the Green Monkey, as in humans, is the a-wave, b-wave, and i-wave. During 30 Hz flicker stimulation, double peaks were often detectable in the b-waves. In these cases, both the amplitudes and implicit times were measured at the first peak. The signal-to-noise ratio was high for all categories, and no extra filter such as a notch filter had to be used, even in single sweep curves. Our mean results obtained in 15 Green Monkeys are summarized in Table 2; the mean amplitudes and latencies of the five standard ISCEV responses are specific to a flash intensity and light adaptation status.

**Table 2:** Responses to standardized electroretinography in Green Monkeys.

Standard response (ISCEV)	a-wave amplitude (µV)	a-wave peak latency (ms)	b-wave amplitude (µV)	b-wave peak latency (ms)	Flash intensity (cd.s.m^−2^)	Adaptation status
Rod response	-	-	88.9±26.6	79.9±6.1	0.0064	Dark
Maximal response	115.1±40.2	14.8±0.7	203.7±52.6	36.7±3.8	2.5	Dark
Oscillatory potential	-	-	60.2±15.5	20.3±0.9	4.4	Dark
Strong flash response	174.9±27.2	9.8±0.4	230.7±40.6	30.8±3.6	10.0	Dark
White flash cone response	22.1±4.5	12.3±1.2	81.5±19.4	27.7±1.5	2.5	Light
30 Hz flicker	-	-	88.9±20.2	24.3±1.0	2.5	Light

Data are reported as mean ± SEM (standard error of the mean).

### ERG responses throughout dark-adaptation

**Figure 3:**
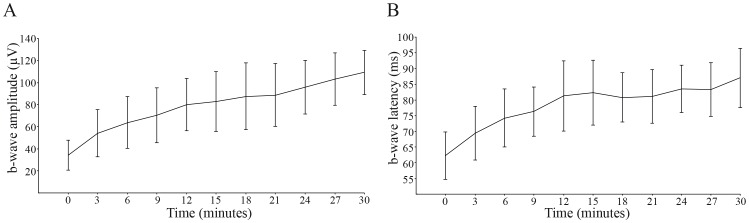
Response versus time functions of b-wave amplitude (A) and latency (B) throughout dark-adaptation elicited at −2.2 log cd.s.m^−2^ (0.0064 cd.s.m^−2^). Each data point indicates average (± SEM) of all 15 monkeys.

ERGs were recorded during the course of dark-adaptation at 3 min intervals over 30 min with a constant stimulus intensity of approximately 0.006 cd.s.m^−2^ (Figure 3). We have not pursued the recordings over 30 minutes based on the human [4] and monkey literature [27]. For example, Bee (2001) reported that in cynomologus monkeys (*Macaca fascicularis*), plateau was reached around 20 minutes.

### Intensity–response function of scotopic and photopic ERG

**Figure 4:**
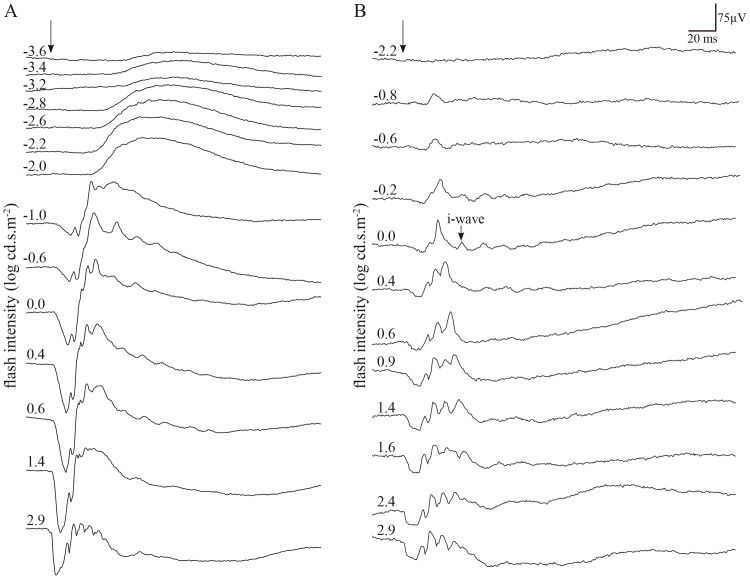
ERG responses to stimuli of increasing flash intensity, from top to bottom, in the dark-adapted eye (A) and in the light-adapted eye (B) of a representative Green Monkey (*Chlorocebus sabaeus*). Vertical arrow indicates flash onset. Horizontal calibration, 20 ms; vertical calibration, 75 µV.

**Figure 5:**
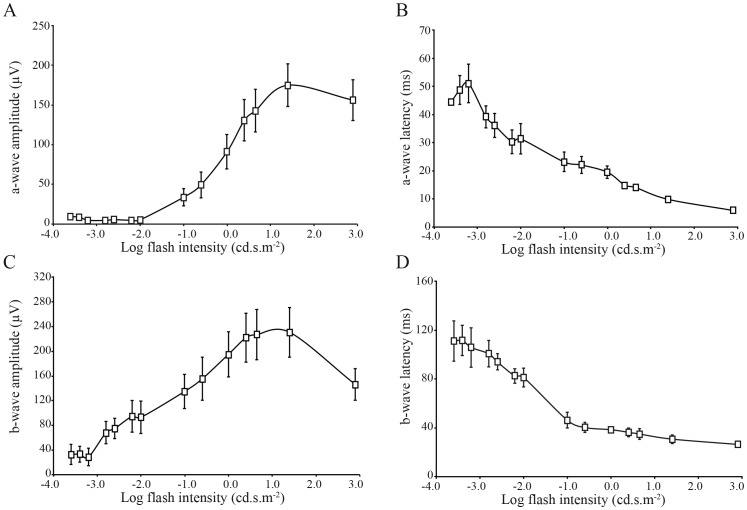
Response versus intensity function for the a-wave amplitude (A), a-wave latency (B), b-wave amplitude (C), and b-wave latency (D) of the scotopic ERG. Each data point indicates average (± SEM) of all 15 monkeys.

**Figure 6:**
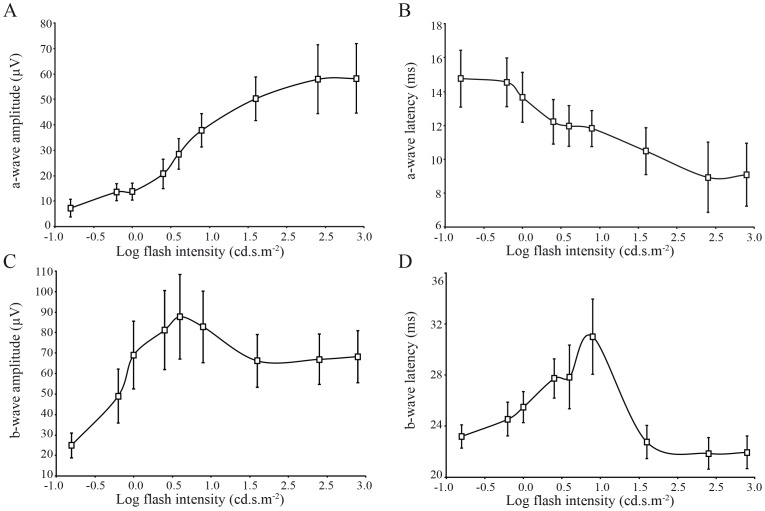
Response versus intensity function for the a-wave amplitude (A), a-wave latency (B), b-wave amplitude (C), and b-wave latency (D) of the photopic ERG under rod-suppressing background illumination (30 cd.m^−2^). Each data point indicates average (± SEM) of all 15 monkeys.

Beyond the ordinary requirements of the ISCEV, ERG responses to stimuli of increasing flash intensity in dark-adapted (Figure 4A) and light-adapted conditions (Figure 4B) were also recorded. Both intensity response series were obtained from the same monkey, and in the same recording session. It can be seen in the figure that the two recording conditions yield ERG responses of different amplitude, timing and morphology. The distribution of full-field ERG amplitudes and implicit times are often asymmetrical, even in large groups of normal monkeys, such that use of statistics based on a normal distribution can be misrepresentative [27]. We used a log transformation of the data to reduce variance. Figure 5 shows the results of the scotopic a-wave and b-wave amplitudes and latency versus log flash intensity (cd.s.m^−2^) functions. It is worthwhile to note a sigmoid curve characterizes the amplitudes as well as the latencies functions in the Green Monkey. Moreover, the b-wave amplitude decreased at the highest intensity of 2.9 log cd.s.m^−2^ with an inter-stimulus interval of 15 seconds (Figure 5A and 5C). The photopic functions are shown in Figure 6. The values given at 0.4 log cd.s.m^−2^ (standard flash) represent the white flash cone response as recommended by the ISCEV. These results are also known from studies on non-human and human primate retinal functions [27], [31].

### The photopic hill effect

In the recordings of light-adapted eyes, the amplitude of the a-wave augments regularly with the gradual increase in intensity of the stimulus, while amplitude of the b-wave first increases to a maximum (V_max_), and finally decreases with presentation of progressively brighter stimuli. This effect has been well demonstrated in humans [32]. The photopic flash ERG of the Green Monkey includes a post b-wave component identified as the i-wave that is best seen using the standard flash (0.0 log cd.s.m^−2^) after light adaptation (Figure 4).

## Discussion

These results provide normative values for the standard ERG protocol in Green Monkeys, with the general finding that full field flash ERG responses in these monkeys are similar to those in humans. We report the 5 responses in Green Monkeys as recommended by ISCEV, which are the standard protocols for ERG in humans [4] and cynomolgus monkeys [27]. The ISCEV consensus standard provides the basis for stable comparison between research laboratories and clinical ERG recordings. However, information about additional stimuli is often necessary for specific applications, such as in considering the higher retinal illuminances for rod responses in human neonates [33], and dark-adapted flicker in retinitis pigmentosa [34]. Throughout the present study, particular attention was paid to IOP and pupil dilatation, with an aim to reduce variability (Table 1). Human and non-human primates are mammals that share similar vascular anatomy of the eyes, and have a macular/foveal region and multiple cone types that offer them high visual acuity and color vision. It is notable that our values of amplitude and latency in Green Monkeys are closer to those in humans [4], relative to corresponding results in cynomolgus monkeys [27], [35], and it may be that the differences are as much attributable to laboratories they are to species differences.

The ERG responses to the standard tests in Green Monkeys were similar to the responses in humans even though the axial length of Green Monkey eyes is a bit lower than humans, i.e. 18 mm in the Green Monkey and 24 mm in humans [36]. In particular, the shape and latency of the curves are highly comparable. We can safely assume that the higher amplitudes found in man [31] are due to the large diameter and larger retinal surface area of the human eye, which have a direct relation with the net electric field and thus on the measured responses. Accordingly, differences in amplitude but not latency have been observed in human subjects with high myopia or small refractive error, as expected due to differences in axial length [37]. In the present study, slight differences in amplitude were occasionally noticed in the other eye, but were in every case within the 10% inter-ocular amplitude differences in normal human subjects [38]. Furthermore, the standard amplitude of OPs was a bit larger than the range in cynomolgus monkeys [27], but lower than those of humans [39]. The implicit times of OPs were within the same range for monkeys and humans.

Full-field stimulation, as employed presently, is the most effective way of eliciting an ERG representative of the entire population of cones and rods in the primate retina [40]. Replicable peak amplitudes and implicit times can therefore be obtained with full-field ERG recordings. Recordings of photopic ERGs are used to assess the functioning of the cone system in humans and animals. As defined above, in response to progressively brighter stimuli, the b-wave of the photopic ERG gradually increases in amplitude, attains a plateau (the maximal b-wave amplitude which is reached for a narrow range of intensities, V_max_), and then rapidly decreases with further increments in the luminance of the flash. This unique luminance–response function was originally termed “the photopic hill” [41]. The photopic hill in the primate ERG results mainly from two factors: the reduction of the ON-component amplitude at higher intensities and the delay in the positive peak of the OFF-component at higher intensities [42]. Scotopic ERGs, on the other hand, are used to evaluate the integrity of the rod system in humans and animals [2], [43].

At about 20 ms after a typical human photopic b-wave, a second positive signal is seen, the i-wave [44]. This feature is common to the photopic ERG of many species except mice and rats [28]. The i-wave amplitudes and latencies in Green Monkeys were similar to those reported previously for most mammals [28]. It is interesting to note that in humans, the amplitude of the i-wave saturates at a dimmer flash intensity than that needed to evoke a b-wave of maximal amplitude [45].

In general, it is important to consider how best to interpret a finding of altered electroretinogram in the clinic and in animal models. Normal scotopic and photopic a-waves indicate normal functioning of rod and cone outer segments. In particular, it has been proposed that the scotopic ERG b-wave is the result of depolarization of ON-bipolar cells [46], [47]. Consequently, a pathological or pharmacological decrease in amplitude of the b-wave of the rod ERG and of the scotopic standard combined ERG might both result from a postsynaptic abnormality in the rod ON-pathway, plausibly due to a postsynaptic abnormality in the cone ON-pathway because it generates this response [46], [48]. The ON-pathway is often considered to influence contrast sensitivity [49], [50]. For instance, impairments of contrast sensitivity are reported clinically in disorders with ON-pathway dysfunction [51], such as melanoma-associated retinopathy [52], congenital stationary night blindness [53].

Specific values for amplitude and b-wave implicit time will necessarily differ between laboratories due to minor variations in recording electrodes, equipment, and protocol, not to mention species differences. Among the various technical factors potentially impacting the ERG amplitudes include contact lens placement, the structural integrity of the corneal surface, pupil size, and even IOP. It is important to consider these factors when interpreting the results. Nevertheless, in order to control for these technical and biological influences, we collected the physiological relevant data before and after the ERG recordings for each monkey, so as to provide a stable basis for comparison in future studies of pharmacology and disease models in the Green Monkey. The present results entailing recordings performed in accordance with the ISCEV, and with the ERG encompassing 30 minutes of dark adaptation correspond very well with similar results obtained in humans. Thus, the Green Monkey promises to serve as an excellent animal model for retinal function testing, for example in toxicity evaluation.

## Supporting Information

Checklist S1 The Animal Research: Reporting In Vivo Experiments (ARRIVE) Guidelines Checklist.
(PDF)
